# Ambulance professionals' experiences of teamwork in the context of a team training programme – a qualitative study

**DOI:** 10.1186/s12873-024-01018-6

**Published:** 2024-07-02

**Authors:** Kjetil Myhr, Randi Ballangrud, Karina Aase, Anne Vifladt

**Affiliations:** 1 Department of Health Sciences Gjøvik, Faculty of Medicine and Health Sciences, Norwegian University of Science and Technology, Gjøvik, 2815 Norway; 2 Department of Acute Medicine, Division of Elverum-Hamar, Innlandet Hospital Trust, Brumunddal, 2381 Norway; 3 Center for Resilience in Healthcare, Faculty of Health Sciences, University of Stavanger, Kjell Arholms Hus, Kjell Arholms Gate 43, Stavanger, 4021 Norway; 4 Department of Research, Innlandet Hospital Trust, Brumunddal, 2381 Norway

**Keywords:** Emergency medical services, Inservice training, Team training, Teamwork, Focus group, Qualitative research, Patient safety

## Abstract

**Background:**

Teamwork in the context of ambulance services exhibits unique characteristics, as this environment involves a small core team that must adapt to a dynamic team structure that involves health care professionals and emergency services. It is essential to acquire a deeper understanding of how ambulance teams operate. Therefore, this study aimed to explore the experiences of ambulance professionals with teamwork and how they were influenced by the implementation of a team training programme.

**Methods:**

A qualitative descriptive study was conducted involving ambulance professionals who took part in focus group interviews carried out both before and after the implementation of a team training program across seven ambulance stations within a Norwegian hospital trust. The data were analysed using reflexive thematic analysis based on a deductive-inductive approach.

**Results:**

Our analysis revealed 15 subthemes that characterised ambulance professionals’ experiences with teamwork and a team training programme, which were organised according to the five main themes of team structure, communication, leadership, situation monitoring, and mutual support. Ambulance professionals’ experiences ranged from the significance of team composition and interpersonal and professional relationships to their preferences regarding different communication styles and the necessity of team leaders within the ambulance service. The team training programme raised awareness of teamwork, while the adoption of teamwork tools was influenced by both individual and contextual factors. The Introduction/Identity, Situation, Background, Assessment and Recommendation (ISBAR) communication tool was identified as the most beneficial aspect of the programme due to its ease of use, which led to improvements in the structure and quality of consultations and information handover.

**Conclusions:**

This study documented the diverse characteristics and preferences associated with teamwork among ambulance professionals, emphasising the particular importance of proficient partnerships in this context. Participation in a team training programme was perceived as a valuable reminder of the significance of teamwork, thus providing a foundation for the enhancement of communication skills.

**Trial registration:**

ClinicalTrials.gov—ID: NCT05244928.

**Supplementary Information:**

The online version contains supplementary material available at 10.1186/s12873-024-01018-6.

## Background

The ambulance service poses unique challenges in terms of teamwork, which has been defined as "the interaction or relationship of two or more health professionals who work interdependently to provide care for patients" [1] (p. 3). A core team of two ambulance professionals (APs) must frequently adapt to a dynamic team structure, as they work closely with other health care professionals and emergency services in a multiteam system [2]. The variety of work tasks, roles, and responsibilities faced by APs creates multiple barriers to effective teamwork, as these professionals care for a diverse patient population with ill-defined and high-acuity diseases and injuries, all while under time pressure. [2].

Fernandez et al. [3] identified the core teamwork processes involved in the ambulance service, including planning, action, reflection, and interpersonal relations, which were all affected by crew familiarity and team cohesion. In a qualitative study conducted by Crowe et al. [4], ambulance professionals highlighted team leadership and team membership as the most important team characteristics of the ambulance service. Furthermore, APs value teamwork skills and prefer proficiency in communication and reflection to medical knowledge and technical skills when assessing professional competence [5].

A great deal of evidence has indicated that team training can improve teamwork in the context of health care [6]; however, efforts to improve teamwork in the ambulance service have mostly been limited to specific training scenarios such as cardiopulmonary resuscitation [7] and trauma [8] rather than encompassing systematic team training over time. This study, which was part of the research project TEAM-AMB [9], implemented the longitudinal team training programme Team Strategies and Tools to Enhance Performance and Patient Safety (TeamSTEPPS) [10]. TeamSTEPPS is an evidence-based team training programme with a curriculum that focuses on team structure and four key skills: communication, leadership, situation monitoring and mutual support. The programme is designed to strengthen patient safety and has proven to be useful in a variety of health care settings [11], resulting in improvements in team competencies such as attitudes, knowledge and performance related to teamwork as well as improved clinical outcomes [12].

There is a paucity of data concerning how APs work in teams and experience teamwork. To improve teamwork in the context of ambulance services, it is crucial to obtain an in-depth understanding of how ambulance teams function and how APs collaborate within the core team of two AP partners and the extended multiteam system. Therefore, this study aimed to explore the experiences of ambulance professionals with teamwork and how they were influenced by the implementation of a team training programme.

## Methods

### Design

This study employed a qualitative descriptive design [13] based on semistructured focus group interviews conducted with APs both before and after the implementation of TeamSTEPPS. The “COnsolidated criteria for REporting Qualitative research” (COREQ) checklist [14] was used to ensure that the study was presented appropriately, see supplementary file 1.

### Setting and sample

This study was conducted at a Norwegian hospital trust that features 129 APs across 7 ambulance stations who participated in the team training programme. The ambulance stations serve a population of approximately 150,000 inhabitants in both urban and rural areas and perform approximately 20,000 missions per year. The APs are licenced emergency medical technicians (EMTs with a four-year vocational high school education), paramedics (EMTs with an additional year of full-time equivalent university education or a three-year university education at the bachelor’s level) and registered nurses (RNs with a three-year university education at the bachelor’s level) who have received additional training and a corresponding certification to work in the ambulance services.

APs with fixed or short-term employment in the seven ambulance stations who participated in the team training programme were eligible for inclusion as participants in the focus groups. Station leaders, locums and trainees were excluded. Participants in the focus groups were identified by the station leaders based on availability, and a convenience sample was invited to participate in this research by the first author (KM) through e-mail and text messages. In total, 21 APs were invited, and 15 APs agreed to participate (Table 1).

**Table 1:** Participants in the focus groups

Categories	Participants (n)	%
Gender		
Female	7	46.7
Male	8	53.3
Experience in years		
≤ 5	4	26.7
6–14	5	33.3
≥ 15	6	40.0
Profession		
Emergency medical technician (EMT)	8	53.3
Paramedic	4	26.7
Registered nurse (RN)	3	20.0

### Intervention

The TeamSTEPPS training programme was implemented in accordance with the TeamSTEPPS implementation guide [10]. A selected group of APs who received master training to serve as TeamSTEPPS instructors formed the “change team” together with the first author (KM). The “change team”, responsible for planing the team training, selected appropriate TeamSTEPPS tools and strategies to fit the needs of the ambulance service (Table 2). The “Training and Implementation” phase started with a full day of introduction to TeamSTEPPS, which was mandatory for all APs and was followed by an initial four months of team training. Each month, the focus of this process was on improving a single ´key skill´ based on the implementation of select strategies and tools.

**Table 2:**
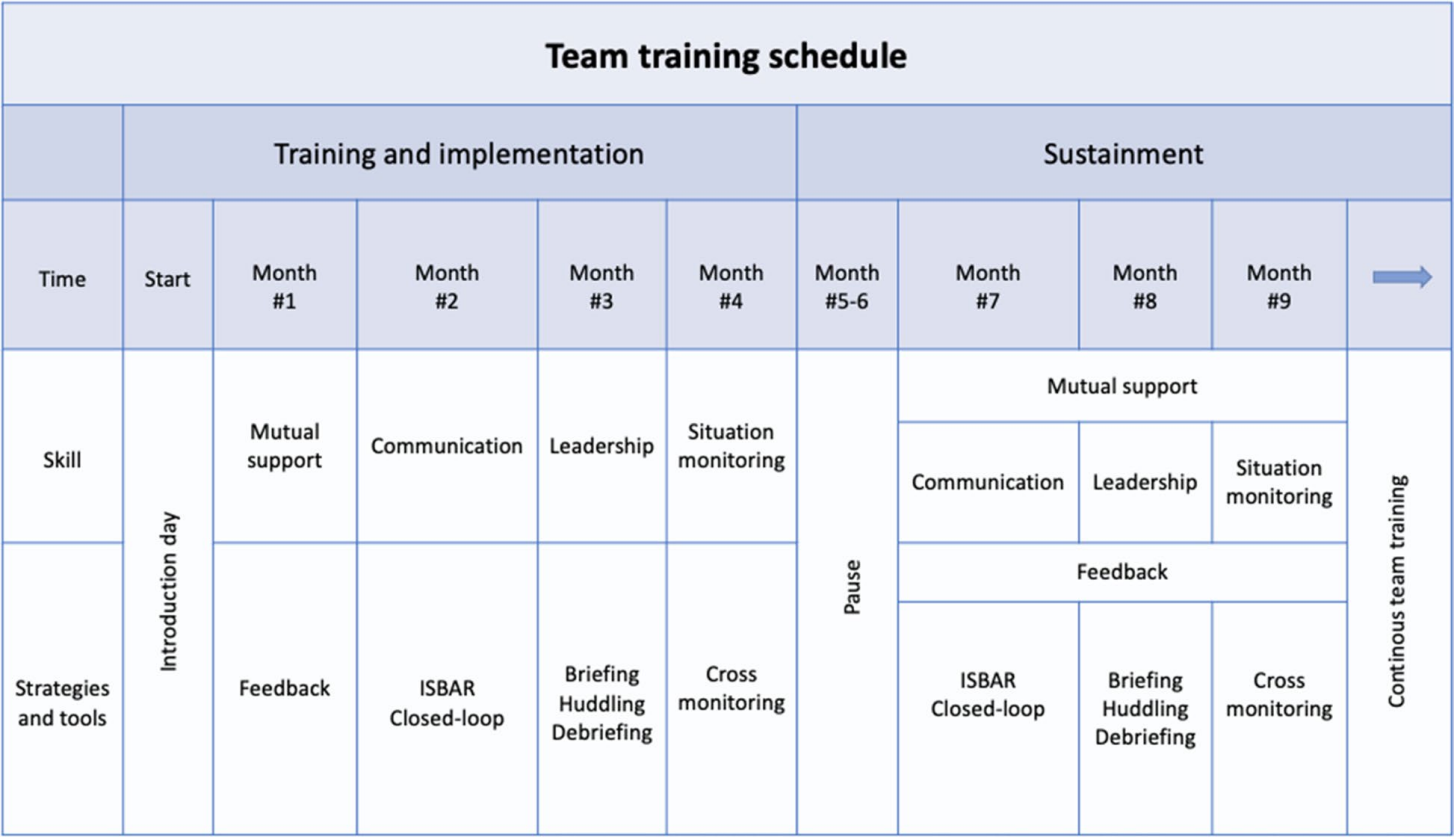
Team training schedule

The following “Sustainment” phase involved a three-month repetition period during which the APs were encouraged to provide verbal feedback to their colleagues concerning the use and development of teamwork skills. Additionally, members of the “change team” sought to incorporate the TeamSTEPPS principles into the regular operations of the ambulance service through established educational activities and simulations.

### Data collection

Focus group interviews were chosen to encourage interaction between the APs and allow for diverse perspectives and reflection on their peers' experiences [15]. Since teamwork is inherently a group activity, it was viewed as a non-sensitive topic suitable for open discussion within a group setting. A semistructured interview guide (supplementary file 2), adapted from a similar in-hospital study [16], was employed to suit the ambulance service setting. The interview guide employed a deductive approach based on the key skills associated with TeamSTEPPS and was pilot tested by reference to four APs who were not included the study sample to ensure clarity. Four focus group interviews were conducted (Table 3), two of which (Focus group 1 and 2) were conducted before the initial four-month training period and two of which (Focus group 3 and 4) were conducted after that period. Table 3 Focus group interviews.

**Table 3:** Focus group interviews

Time	Before team training		After team training	
Group interview	Focus group 1	Focus group 2	Focus group 3	Focus group 4
Participants* (n)	5	5	6	4
Length of interview (min)	81	67	83	77

^*^Five participants participated in interviews both before and after the team training programme

The interviews were conducted at an ambulance station, and the researchers were involved as a moderator (KM) and as an observer (AV) who took field notes. Each interview started by specifying the aim of the study and encouraging the participants to speak freely. At the end of each interview, the observer verbally summarised the main topics to facilitate clarification and elaboration. The interviews were audio recorded using a dictaphone application [17] and subsequently transcribed verbatim (KM) and anonymised. After each interview, the quality and breadth of the discussions were evaluated. It was determined that the focus groups had provided a sufficiently rich dialogue to analyse the data and address the aim of the study after four interviews, thus reaching appropriate information power [18].

### Data analysis

This study employed an analytical approach based on the principles of reflexive thematic analysis (RTA) [19, 20]. RTA is a flexible and extensively researched method for analysing qualitative data and exhibits a particular focus on the fact that the researcher, including his or her background and preconceptions, is an integral part of the qualitative research process. Although this approach is structured and features well-outlined sequential steps of analysis, it is a recursive process rather than a linear process [21].

Based on the aim of the study and the characteristics of the data, a deductive-inductive thematic structure for the initial analysis was employed based on the TeamSTEPPS framework [10], as shown in Table 4. The NVivo software (QSR International, 2020) was used to structure and document the research process and divided the data into two data sets, i.e., one before and one after the team training programme. Analysis of both data sets started with an initial process of familiarisation that involved listening to the audio recordings, reading the transcripts, and critically considering the context and meaning of the data before the authors met to discuss their preliminary impressions and reflections.

**Table 4:** Description of the TeamSTEPPS framework [10]

	TeamSTEPPS framework
Team structure	Team structure refers to the composition of the team and the interactions among the patient, caregivers, and other figures who play a supportive role (family, friends, administration, ancillary services) in terms of their profession(s), competencies, roles and responsibilities
Leadership	The ability to maximise the activities of team members by ensuring that team actions are understood, changes in information are shared and team members have the necessary resources
Communication	The structured process by which information is exchanged among team members clearly and accurately
Situation monitoring	The process of actively scanning and assessing situational elements to gain information, to obtain a better understanding, or to maintain the awareness necessary to support team functioning
Mutual support	The ability to anticipate and support team members’ needs based on accurate knowledge of their responsibilities and workload

The data were coded (KM) and recoded (KM/RB/KA/AV) in accordance with the five TeamSTEPPS themes. Materials that exhibited possible relevance but no obvious affiliation with a theme were coded as such and subsequently reviewed to avoid premature analytical closure. The codes were then either sorted into one of the existing themes or discarded if believed to be insignificant.

Thereafter, an inductive approach was used to identify patterns of meaning within the codes pertaining to each theme to identify possible subthemes. Again, codes that lacked a clear and structured initial meaning were reviewed and either sorted into a new or existing subtheme or discarded. Ultimately, an iterative process involving discussions among the authors led to the development of the subthemes. An example of the analysis process is shown in Table 5.

**Table 5:**
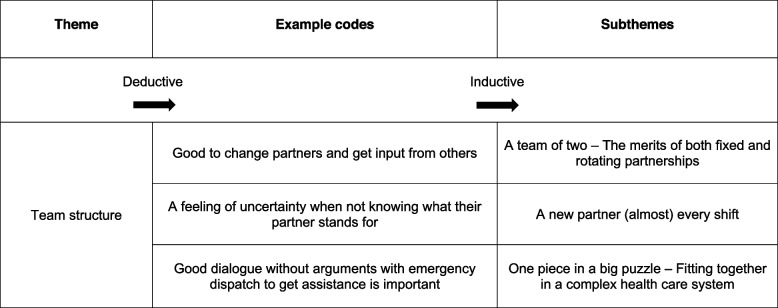
Example of the analysis process

## Results

The results are presented thematically with subthemes both before and after the team training, as shown in Table 6. Quotations are labelled to indicate the AP who made the statement in question (ranging from AP1 to AP15) and the focus group interview from which the quotation was drawn (Focus group 1 to 4).

**Table 6:** Overview of themes and subthemes before and after team training

	**Themes**	**Subthemes**
Before team training	Team structure	A team of two – The merits of both fixed and rotating partnerships A new partner (almost) every shift One piece in a big puzzle – Fitting together in a complex health care system
	Communication	Information exchange between AP partners Communication across services – Tone and timing
	Leadership	“Do we really need a team leader?” Before, during and after – Leadership as an ongoing process
	Situation monitoring	Checking and rechecking – Monitoring the patient and your partner Barriers to situational awareness across a multiteam system
	Mutual support	Cultivating relationships promotes a supportive environment
After team training	Team structure	Team training creates common ground
	Communication	Communication tools promote decision-making and save time
	Leadership	Mixed experiences with training leadership skills
	Situation monitoring	Good intentions fall short – A mismatch between protocol and reality
	Mutual support	Team training with the goal of improving feedback– A small step in the right direction

### Before team training

The 10 subthemes that represent APs experiences with teamwork before the team training programme are presented within the five themes based on the TeamSTEPPS framework.

### Team structure

#### A team of two – The merits of both fixed and rotating partnerships

The APs reported different perspectives on the ways in which ambulance teams should be structured. Fixed partnerships were preferred by most APs, provided that they were comfortable working with the other AP. Working with and establishing a relationship with one’s partner over time creates a feeling of safety and mutual trust that is crucial for teamwork.

*"It's very comfortable to drive together with the colleagues with whom we have worked over the years. We work better, and things run more smoothly."*

*(AP5 Focus group 1)*

Another beneficial result of a well-functioning fixed partnership was the ability to attain a state of flow, in which context the APs anticipate each other’s needs and actions, and their behaviour becomes automated. In contrast, some APs argued that a fixed partnership might limit new input and therefore impede professional development. Additionally, when working with less familiar partners, one might be more attentive and compelled to verbalise one’s thoughts and ideas.

#### A new partner (almost) every shift

Frequent use of locums, especially during the summer holiday, influenced team structure and increased the workload and responsibility faced by more experienced APs.

*"The problem is not rotating with the people in this room [participants in the focus group interview]. The problem is the frequent use of locums.”* *(AP3 Focus group 1)*

Locums could lack qualifications that would otherwise be mandatory for regular practice, such as emergency response driving or medication handling; alternatively, they might be unfamiliar with the locations in which equipment is stored or ways of communicating with other emergency services.

*"[With a very sick patient,] my place is perhaps in the back with the patient. He [the locum] can drive the ambulance, of course, but not during emergency call-out missions." (AP5 Focus group 1)*

The uncertainty concerning the competence of their new partner and how they address critical situations was experienced as a burden by the APs and exhibited the potential to increase their stress levels.

#### One piece in a big puzzle – Fitting together in a complex health care system

APs frequently operate in the context of extended teams that include participants from other professions, who might have different mindsets and priorities. A source of frustration for the APs was the lack of comprehension of their actions or inactions on the part of emergency room staff. The APs highlighted that due to constraints in the work environment as well as limited time and resources, certain tasks or procedures might not have been performed (e.g., the placement of an intravenous cannula) prior to arrival at the emergency department. As parts of a multiteam system, the APs recognized the need to understand the differences between the prehospital work environment and the in-hospital work environment to avoid conflict.

In situations in which the assistance of other health care professionals or emergency services was necessary, the APs valued colleagues who introduced themselves and asked how they could help rather than jumping into the action, taking over, or ignoring the protocols of the ambulance service. Members of the fire department were identified as experts in teamwork in terms of how they offered assistance, communicated on the scene, and avoided interruptions.

*"They [the fire department] are extremely good at this [teamwork]... Perhaps it is because they have sufficient time for it [communication training]. We are constantly driving; we do not have enough time." (AP3 Focus group 1)*

### Communication

#### Information exchange between AP partners

During a shift, APs exchange an extensive amount of information with their partner, and they recognized the fact that differences in communication quality might impair or facilitate teamwork. Partners who use similar terminology and phrases connect more easily, communicate more efficiently, and thereby establish a sense of trust in their experience and knowledge in their field. Furthermore, the APs emphasised the role of communication in the process of establishing personal relationships with their partner.

*"You have to be able to talk and be confident with each other. We must get to know each other." (AP10 Focus group 2)*

Well-established AP partnerships might feature less explicit communication as workflows and procedures become automated, such that the APs come to understand each other's actions and intentions without the need for verbal cues. Other APs preferred more dialogue as a way of preventing uncertainty and ensuring shared situational awareness.

#### Communication across services – Tone and timing

The APs preferred open and friendly initial communication in their interactions with other services. The manner in which one is greeted sets the tone for future teamwork.

*"Or when you are met with 'What are you doing here?' in the emergency department. Then, you feel.... you feel ashamed. Especially on behalf of the patient." (AP8 Focus group 2)*

The APs communicated frequently over the radio, which could be experienced as a source of frustration when the receiver did not acknowledge what was said, when the communication involved the “closed-loop” approach, or when excessive unnecessary information was shared, leading to clutter or overload. Furthermore, the APs were frequently interrupted and asked to provide status updates, sometimes repeatedly, which could disrupt their workflow and lead to annoyance on the part of the APs.

### Leadership

#### Do we really need a team leader?

The ambulance service taking part in this study had defined in its protocol a leadership role for major incidents, e.g., traffic accidents, where one AP is responsible for obtaining an overview of the situation and facilitating cooperation with other emergency services. Beyond these situations, the APs reported that they rarely identified themselves as team leaders.

The APs indicated that they usually switched roles after every mission regardless of seniority and qualifications, and they noted that both partners performed leadership tasks. The AP who drove the ambulance was responsible for collecting the patient’s medical history and examining the patient, while their partner prepared equipment and medications and collected supplemental information from bystanders. The APs had different opinions concerning the need for a team leader.

*"[A team leader who has] an overview of what is going on may be useful.” (AP4 Focus group 1)*

*"When we work in a partnership of two, we do not really need a leader; there is no need to appoint a leader... It goes automatically, and often the person who has the patient sort of takes control." (AP12 Focus group 2)*

In cases involving serious incidents, at which more than one ambulance and possibly additional emergency services are present, the APs highlighted the possible benefits of a designated leader. Ideally, this leader would be an AP who could focus on obtaining an overview of the situation without being distracted by the need to perform practical tasks. However, this situation was rarely observed, as high-demand situations often require APs to perform hands-on tasks.

*"It came up again after the cardiac arrests that we should have had an ILH [team leader]. We have said it for quite some time; everyone has said that we should have had an ILH [team leader] who could provide more order and even more structure, but no one does it." (AP2 Focus group 1)*

*“The same chaos every time”* *(AP5 Focus group 1)*

#### Before, during and after – Leadership as an ongoing process

Although APs did not necessarily view themselves as leaders, they frequently employed leadership strategies such as planning and shared decision-making that guided team efforts. The senior APs recognised their ability to serve as role models and were aware of the fact that their behaviour was particularly important when working with new partners.

*"I do expect you to speak up if there is anything you are wondering about. Do not be afraid to ask, as there is no such thing as a stupid question. And I will also ask you if I have any questions, is what I would say [to the locum]. To lower their guard, in a way, so that they do not think it is too difficult to ask about anything." (AP8 Focus group 2)*

The APs agreed that planning while traveling to a patient was beneficial. They indicated that ensuring that the APs exhibited similar ways of thinking about the mission, alongside the ambulance service protocols, formed the basis of an action plan. Planning was viewed as particularly important when working with new partners or trainees.

*"We most certainly have room for improvement when it comes to planning on our way out [to a mission], at least occasionally, as sometimes we plan and sometimes we do not. And we see that things might go astray if we do not plan ahead. I have to admit that it has happened sometimes." (AP4 Focus group 1)*

After the initial patient assessment, the APs employed leadership strategies by engaging in discussions concerning the next action that needed to be taken and, in cases in which the answer was not obvious, where the patient should be transported. The APs preferred partners who shared their ways of thinking and engaged in open conversations concerning how the team should care for the patient.

*"And maybe we could talk about the mission afterwards. What was good? What was bad? What could we have done differently? Perhaps there is someone with whom you do that more than others." (AP10 Focus group 2)*

After a mission, whether in the ambulance or after returning to the station, the APs often discussed the case and the care they provided. These discussions mostly took the form of informal conversations, but they tended to be more detailed in cases involving trainees, as the APs recognised debriefing to be a vital part of trainees’ education. While formal debriefing sessions did occur, they were rare and usually occurred only after very serious incidents involving multiple services. Compared to other emergency services, the APs indicated that they had less time to debrief properly, as they were frequently required for new missions that could not wait.

### Situation monitoring

#### Checking and rechecking – Monitoring the patient and your partner

The APs explained that working with a partner who was new or lacked experience could be difficult because they were required to devote time and effort to the tasks of supervising and monitoring their partner's work. These responsibilities could divert their attention from the patient and their own responsibilities, and in stressful situations, they could lose sight of the overall picture they needed to maintain situational awareness. However, while monitoring their partner might be burdensome, the APs also recognised their role as a safety net.

*"If we have a really sick patient, it is easy to get lost in your own bubble. There might be things you forget along the way, and then it is good to have a partner who is involved in the same line of thinking" (AP5 Focus group 1).*

#### Barriers to situational awareness across a multiteam system

The APs indicated that some colleagues, especially in-hospital staff, lacked insight into the environment and context in which APs worked.

*"The staff at the emergency department do not understand how prehospital work is conducted, and we might not understand why the emergency department is nagging us." (AP3 Focus group 1)*

Achieving shared situational awareness within the multiteam system was recognised as important by the APs, but conveying information about critically ill patients could be challenging. The APs reported how multiple parties, such as emergency dispatch workers, members of the emergency department or air ambulance physicians, often request status updates while the APs might be occupied with patient care. In particular, when communicating via radio, important details concerning the patient might be lost or misunderstood.

### Mutual support

#### Cultivating relationships can promote a supportive environment

The APs recognised the importance of trusting their colleagues, recognising that their support is paramount when APs are required to address challenging situations and to feel safe while doing so. APs spend a great deal of time together, and being able to discuss issues beyond the context of work and establishing personal relationships can foster teamwork. Even after missions, the APs felt that debriefing a difficult case was easier with partners whom they trusted and with whom they had personal bonds.

*"We are like a small family in a way. If we do not get along with each other and we cannot talk to each other, then things will not work out on the mission either. We need to be able to get along when things are quiet as well" (AP10 Focus group 2)*

APs' workflow depended on practical support, and they often operated jointly rather than working independently on separate assignments. The APs considered patient care to be a team effort and regularly intercepted, complemented, or took responsibility for other’s tasks. Familiar and experienced APs could anticipate the needs of their patients and partners and prepared the appropriate equipment and medications ahead of time.

*"You try to bring out the best in your partner, and in doing that, there is a big difference between someone who is experienced and someone who is not." (AP5 Focus group 1)*

### After team training

The five subthemes that represent APs experiences with teamwork after the team training programme are presented within the five themes based on the TeamSTEPPS framework.

### Team structure

#### Team training creates common ground

The APs experienced team training as creating a mutual structure that could guide teamwork. The individuals with whom they were partnered were less important when they possessed the same teamwork skills.

*"For me as a locum working with many different colleagues, it's good that things are put into order. Personally, I like having a system. I need it to function and collaborate with everyone." (AP14 Focus group 4)*

When working with locums who had not participated in the team training programme, some APs felt that they were discouraged from using their newly acquired teamwork skills, while others continued their focus on teamwork as a way to model this behaviour for their new colleagues.

*"We can use it [the skills from team training] as a structure in the beginning so that they [the locums] can learn how to use those types of tools. Perhaps it is easier for them to get to know the structure if we keep going." (AP2 Focus group 3)*

### Communication

#### Communication tools promote decision-making and save time

The APs reported widespread use of the communication tools “closed-loop” and “Introduction/Identity, Situation, Background, Assessment and Recommendation” (ISBAR) after the team training programme. A printed version of the ISBAR approach that was located on the back of their identification cards served as a useful and easily accessible reminder according to the APs.

*"I think it has worked very well for using ISBAR, and I feel I am getting someone on the other end who actually bothers to listen to me. Previously, there may have been a bit too much back and forth without any structure." (AP6 Focus group 3)*

The APs indicated that by using ISBAR, consultation with cooperating health care professionals could decrease the time required to provide definite care for the patient. For example, this approach could enable APs to avoid unnecessary and time-consuming visits to the general practitioner’s office or the casualty clinic, instead focusing on providing direct transportation to the hospital. Furthermore, when they used ISBAR to convey information, the APs perceived that they sounded more professional and that the person on the receiving end had more trust in their decision-making.

While the “closed-loop” and ISBAR approaches were described as easy to use and associated with immediate recognizable benefits, they still required effort to change behaviour, and some APs returned to their old accustomed habits when consulting other health care services.

### Leadership

#### Mixed experiences with training leadership skills

The APs were divided in terms of their impressions regarding how the team training programme had affected team leadership in their service. One group valued leadership skills highly, viewing them as a prerequisite for team functioning, and indicated that most of their colleagues were eager to participate in acts of leadership following the team training. Using checklists, which are easily visible in ambulances, they initiated briefing and debriefing (leadership strategies) more frequently. For some APs, this programme made it easier to engage in such activities, as they could refer to the training programme as their reason for adopting this approach.

*"My impression is that it [playing the role of team leader] has been normalised and that we have agreed that this is how it should be as we can benefit from it. We have become better at pointing to each other [as team leaders] and daring to stand out in front of everyone else." (AP14 Focus group 4)*

The other group, however, identified leadership as the most difficult teamwork skill. They highlighted the difficulties they faced with regard to being in charge and telling their partners what to do, and they indicated that sticking their necks out by serving as a leader broke with the cultural norms of the ambulance service. They also highlighted the fact that their partnerships usually functioned adequately without the need for a designated leader and therefore did not understand the need for change in this respect.

In general, both groups agreed that they had become more aware of the role and impact of leadership after participating in the team training programme. Following their initial enthusiasm, a decrease in the use of the leadership strategies was described by the APs as the focus of the team training shifted. Some APs reported that they probably used the tools frequently without being aware of doing so.

### Situation monitoring

#### Good intentions fall short – A mismatch between protocol and reality

The medication administration process was identified as an area of specific importance with regard to situation monitoring in the team training programme. Cross-monitoring and double control of medication administration and examples of how the process should be conducted (according to local protocols) were provided. While the APs recognised the benefits of monitoring each other with regard to, they described working in an environment in which it was often not possible to adhere to the desired standards, such as during transport or when their partner was occupied with other tasks.

*"It [the team training] has made me think more about what we are doing, but there are some challenges and a lot that is difficult to change... I certainly think there is a somewhat stricter framework [in the team training] for medication management than perhaps what we have practised previously. A lot more double controls than I am used to." (AP6 Focus group 3)*

The incongruity between what the APs were told to do and what they felt was actually feasible decreased their interest in continuing to train this particular skill. When prompted, however, the APs reported that after the team training programme, they were more aware of their environment and better able to achieve shared situational awareness with their partners. They highlighted the ways in which the use of tools drawn from other teamwork skills, such as the “closed-loop” and “huddling” approaches, helped them obtain and regain a mutual understanding of challenging situations.

### Mutual support

#### Team training with the goal of improving feedback – A small step in the right direction

The APs had distinct views concerning the ways in which the team training programme had affected their ability to support their colleagues and offer feedback. Differences in personality were identified as the main reason why certain APs were more willing to give and receive feedback. Some APs were encouraged by the team training programme and used it as an excuse to provide feedback, while others felt that doing so was as difficult as it had been before the training programme.

*"I find it easier to pick up the thread after the mission [after the team training], yet it is just as hard to give feedback. At least for me, since I am new at this job, it feels like ‘should I give feedback to AP13 who has been working here for 10 years?’ That is somehow hard to do." (AP15 Focus group 4)*

The APs agreed about the value of supporting each other through feedback and highlighted the ability of this approach to foster professional development; however, fear of hurting their colleagues often prevented them from communicating information that could be perceived as criticism. After reflecting on the difficulties of feedback, one AP made the following statement:

*"Yesterday, I was praised for several things, and I took it to heart much more and was happier with the feedback than I might have been before. So, that is probably what I am left with the most [after the team training], that I think more about the fact that it was really nice that AP7 gave me feedback." (AP6 Focus group 3)*

## Discussion

In this study, we described the experiences of APs with teamwork. Our results highlight the importance of team composition, the challenges associated with APs’ work on teams, and the impact of team training on the development of teamwork skills.

The APs in our study clearly preferred to work with familiar partners and described how staff turnover and the frequent use of locums were detrimental to team performance and increased their stress levels. The intimate work relationships among APs and the existence of joint downtime between missions is a prominent characteristic of the ambulance service, and one could argue that a personal connection with one’s partner is of particular importance in this context [22, 23]. Working most of one’s shifts with the same partner or rotating partners within a small group of colleagues with whom one familiar (alongside occasional shifts to new partner, which can allow one to remain attentive and receive new input) would be optimal according to the APs. Previous research has shown that familiarity among health care professionals is associated with improved outcomes for surgical patients [24] and lower mortality in intensive care settings [25]. In the study conducted by Cottrell et al. [26], familiarity among APs was shown to improve communication and the safety of paediatric patient care. Although Patterson et al. [27] reported a lower incidence of workplace injury among APs who were familiar with one another, a subsequent study did not support this hypothesis [28]. Overall, crew familiarity is an important factor with regard to interpersonal teamwork processes in the ambulance service [3], thus making it crucial to allocate time and resources to the task of creating a work schedule that pairs APs appropriately.

Communication was a reoccurring topic across the different subthemes associated with APs’ experiences of teamwork. The APs recognised the value of communication but had different preferences regarding its style and frequency. Some APs described well-working partnerships in which in-action verbal communication was less frequent and behaviour was mostly automated. Others, including experienced APs, preferred explicit communication involving ongoing discussions about patient care as a means of ensuring shared situational awareness. The APs agreed that verbalising one’s thoughts was more important when working with locums or other health care professionals within the wider multiteam system. The APs also reported that team performance decreased when communication became too frequent or mistimed and that the quality of communication was more important than its frequency, as was reported in the meta-analysis conducted by Marlow et al. [29].

In complex, high-acuity cases, APs highlighted the need for a team leader to guide team efforts. However, few APs viewed themselves as leaders within the team. The ambulance service exhibits strong cultural norms [22], and the ingrained concept of an equal partnership could explain the lack of such a leadership identity among APs. Rather, with occasional exceptions, the APs described a nonhierarchical structure in which both APs performed acts related to leadership, such as planning and decision-making. Furthermore, the APs indicated that the omission of the planning process could lead to mission failure and suboptimal care. This finding is in line with the principal finding reported by Crowe et al. [4], who identified planning and creating an action plan as the most important components of team leadership in the ambulance service.

By working closely together, APs often develop personal, almost familial bonds, and feeling safe with their partner was identified as crucial by the APs included in this study. The mutual dependency and joint downtime between missions observed in this context are somewhat unique to the ambulance service, and establishing good interpersonal relationships is vital for appropriate partnerships and quality in the context of teamwork [23]. According to the APs, it was the responsibility of the more experienced AP to establish an environment in which their partner felt safe to ask questions and make mistakes. Research has shown that teams that are characterized by psychological safety are more likely to improve as a result of team training and to exhibit higher levels of team performance [30, 31]. However, challenges might occur when the team is composed of APs who have significantly different levels of experience, as previous research has reported that novice APs may experience increased stress levels when working alongside more senior colleagues [23].

A total of seven tools were implemented during the team training programme on which this study focuses. When both APs used the tools, the importance of working with a well-known partner decreased, and teamwork became easier. Participating in similar team training offered APs a recognizable structure for teamwork and has been shown to connect health care workers even across professions [16].

The communication tool ISBAR was reported to be easy to use and to offer immediate benefits, such as by decreasing the time required to provide definite care for certain patients. The structured approach to information exchange, which was easily accessible for reference on the back of the APs’ identity cards, was successfully adopted by most APs. To date, ISBAR represents the most widely implemented communication tool in health care and has been endorsed by the World Health Organisation (WHO) and numerous other organisations [32].

While some APs embraced the focus on leadership skills exhibited by the team training programme, others neglected this area of focus. The seven ambulance stations were organised into two units based on their location, and the engagement of the APs in developing leadership skills exhibited notable differences between these units, despite the fact that their members participated in the same training. Cultural differences and local contexts could serve as a partial explanation of this situation, as could the effects of social contagion, according to which professionals adopt the attitudes and behaviours of prominent individuals within their group [33].

The team training programme’s attempt to improve the medication administration process by focusing on situation monitoring was less successful. The examples provided to the APs regarding how to double check medications were perceived as overly cumbersome and not feasible in regular practice and were therefore disregarded by many APs. Given the practical difficulties of double checking medications in a “textbook” manner in the ambulance service as well as the lack of evidence indicating that this approach actually reduces errors in medication administration [34], decision-makers should take these practical contingencies into account in their attempts to improve medication safety in the ambulance service.

### Limitations

Several limitations in our study merit acknowledgment. Firstly, due to availability, the composition of the focus groups changed from before to after the team training. Five new individuals took part in focus groups 3 and 4, two of whom had not fully participated in the team training, limiting their experience with the programme and potentially the credibility of our results [35]. Additionally, contextual factors such as high turnover rates among APs and budget cuts in the ambulance service influenced the team training programme, affecting the transferability of our findings to other settings.

Furthermore, the authors did not have any personal or professional relationships with the participants in the focus groups; however, KM’s role as a physician and coordinator of the team training programme could have influenced the interview and analysis process and therefore the confirmability of the study [35]. Throughout the study, KM tried to remain cognizant of how his role, prior beliefs and vested interests affected the research process. Lastly, the qualitative data from this study was used to explore APs' experiences with teamwork and a team training programme, and should not, by itself, be used to assess the efficacy of the intervention. Subsequent publications using quantitative data will explore this further.

## Conclusions

This study showed that APs generally experience teamwork as effective but have varied preferences regarding its practice. The APs preferred working with the same partner over time, and developing relationships, both professional and interpersonal, was important for teamwork. The implementation of a team training programme contributed to greater awareness of the importance of teamwork, mitigated disparities in professional behaviour and provided the APs with tools and strategies that were adapted to a varying degree.

Future research should investigate different approaches to teamwork among APs, especially by exploring team leadership through observational studies. Additionally, assessing the long-term impacts of team training and employing qualitative methods to further refine team training programmes can facilitate improvement work in the ambulance services.

### Supplementary Information

Supplementary Material 1.

Supplementary Material 2.

## Data Availability

The data is not publicly available due to the participants’ consent agreement and confidentiality policy. Parts of the dataset, transcribed in Norwegian, can be made available upon reasonable request to the corresponding author.

## Abbreviations

AP: Ambulance professional
ISBAR: Introduction/Identity, Situation, Background, Assessment and Recommendation
TeamSTEPPS: Team Strategies and Tools to Enhance Performance and Patient Safety
