# Supplementary material for: Ambulance professionals' experiences of teamwork in the context of a team training programme – a qualitative study
Source: BMC Emerg Med. 2024 Jul 2;24:108. doi: 10.1186/s12873-024-01018-6 (PMC11218233; doi:10.1186/s12873-024-01018-6)
Supplement: Supplementary file 2 — Supplementary Material 2. [file 12873_2024_1018_MOESM2_ESM.docx]

**Semistructured interview guide**

**Team Training and Medication Administration in Ambulance Services - TEAM-AMB**

**a "Human Factors" approach**

**1st interview**

Firstly, we would like to thank you for participating in this focus group interview today. The study we are conducting aims to describe ambulance personnel's experiences with teamwork (and eventually experiences with the introduction of a team training program). As you are aware, parts of the ambulance service in Innlandet will undergo a team training program. We wish to interview you before and after the implementation of the team training, once now and again in approximately five months.

In the ambulance service, you work together in teams around the patient, and teamwork is described as collaboration among two or more healthcare professionals who work interdependently to provide treatment and care to patients. One works in both interdisciplinary teams (with other professional groups) and monodisciplinary teams (only one professional group).

With this interview, we want to learn more about how you experience teamwork within the ambulance service and in relation to other collaborative groups (such as primary and specialist healthcare services, police/fire departments, and others) and what you think about participating in a team training program. It is natural to inquire about:

- Who you are and what kind of work experience you have?
- How (in what way) do you work together as ambulance personnel in a team?

(Feel free to describe how you work together during a shift)

- - What characterizes good teamwork among ambulance personnel?
  - What challenges might you face regarding teamwork among ambulance personnel? (e.g., with severely injured/critically ill patients)
  - What promotes good teamwork?
  - What hinders good teamwork?
- Team Training
  - What are your thoughts on participating in a team training program?
  - How do you think a team training program can contribute to promoting quality and patient safety in ambulance services?
- How (in what way) do you work in teams with other healthcare personnel? (nurses and doctors in primary and specialist healthcare services) •
  - What characterizes good teamwork?
  - What challenges might you encounter? (e.g., with severely injured/critically ill patients or transferring information)
  - What promotes good teamwork?
  - What hinders good teamwork?

**2nd interview**

Firstly, we would like to thank you for participating in this focus group interview today. The study we are conducting aims to describe ambulance personnel's experiences with teamwork and experiences with the implementation of a team training program. As you know, parts of the ambulance service in Innlandet are undergoing a team training program called TeamSTEPPS. The overarching goal of the TeamSTEPPS project is patient safety:

- Improved patient safety culture
- Fewer adverse events
- Good quality and satisfied patients

In the ambulance service, you work together in teams around the patient, and teamwork is described as collaboration among two or more healthcare professionals who work interdependently to provide treatment and care to patients. One works in both interdisciplinary teams (with other professional groups) and monodisciplinary teams (only one professional group).

With this final interview, we want to learn more about how you experience teamwork within the ambulance service and in relation to other collaborative groups (such as primary and specialist healthcare services, police/fire departments, and others) and what you think about having undergone a team training program.

Teamwork Program

- How do you feel about participating in TeamSTEPPS?
- How did you experience the introduction day to TeamSTEPPS?
- How do you experience the ongoing implementation of TeamSTEPPS in the department?
- Do you feel that participation in the team training program changes attitudes towards patient safety in the ambulance service?

Team Structure

- Is awareness created about working in a team?
- What hinders achieving good teamwork in the ambulance service?
- Are patients included in their own treatment and as part of the team?

Communication

- To what extent has TeamSTEPPS created awareness about the importance of good communication?
- How do you experience communication in the department now?
- Do you experience communication challenges in the department? If yes, can you describe these?
- What measures (tools, strategies) have been implemented so far to promote communication in the team?
- What have you improved upon?
- What needs further work?

Leadership

- To what extent is awareness created about leadership in the team?
- What does good team leadership entail?
- What measures (tools, strategies) have been implemented so far to promote leadership in the team?
- What have you improved upon?
- What needs further work?

Situation Monitoring

Situation monitoring is a method that makes team members aware of what is happening around them related to the patient, the team, surroundings, and progress towards goals.

- To what extent is awareness created about situation monitoring?
- How does this function in the teams you are involved in?
- How can a common understanding be established within a team, and how can this be developed?
- What have you improved upon?
- What needs further work?

Mutual Support

- To what extent is awareness created about mutual support?
- How does mutual support affect team processes?
- Can you say something about what can promote mutual support (e.g., helping each other with tasks, providing feedback) within a team?
- What have you improved upon?
- What needs further work?
